# In vitro effects of PI3K/mTOR inhibition in canine hemangiosarcoma

**DOI:** 10.1371/journal.pone.0200634

**Published:** 2018-07-16

**Authors:** Alex A. Pyuen, Travis Meuten, Barbara J. Rose, Douglas H. Thamm

**Affiliations:** 1 Flint Animal Cancer Center, College of Veterinary Medicine and Biomedical Sciences, Colorado State University, Fort Collins, CO, United States of America; 2 Cell and Molecular Biology Graduate Program, Colorado State University, Fort Collins, CO, United States of America; 3 Comprehensive Cancer Center, University of Colorado, Aurora, CO, United States of America; Bauer Research Foundation, UNITED STATES

## Abstract

While extremely rare in humans, hemangiosarcoma (HSA) accounts for nearly 2% of canine neoplasia, and is characterized by both aggressive local growth/invasion and a high rate of metastasis. Both canine and human HSA exhibit sustained aberrant PI3K/Akt/mTOR pathway signaling. The purpose of this study was to examine the *in vitro* effects of a novel dual PI3K/mTOR inhibitor, VDC-597, in three canine HSA cell lines (DEN-, CIN-, and SB-HSA). VDC-597 suppressed activation of both Akt and 4eBP1 in canine HSA cells in a dose-dependent fashion, with an IC50 of approximately 0.3 uM, a concentration predicted to be clinically achievable based on preliminary early-phase canine and human studies. VDC-597 dose-dependently reduced proliferation, migration, and vascular endothelial growth factor production in HSA cells, while promoting tumor cell apoptosis. VDC-597 demonstrated additive antiproliferative effects when combined with doxorubicin. These results suggest that inhibitors of the PI3K/mTOR pathway may act against multiple components of the neoplastic process, including proliferation/apoptosis, chemosensitivity, migration, and angiogenesis, and justify the evaluation of PI3K/mTOR inhibitors in canine, and potentially human, HSA.

## Introduction

Canine hemangiosarcoma (HSA) is an aggressive neoplasm derived from endothelial cells or hematopoietic precursors that accounts for nearly 2% of all cancer diagnosed in dogs [1, 2]. The most common sites of involvement are the spleen, skin and subcutaneous tissues, and the heart [3]. Current standard of care treatment involves surgical resection (if possible) followed by doxorubicin (DOX)-based chemotherapy. Regardless of the treatment protocol, the median postsurgical survival time for dogs with visceral HSA is less than 6 months [4]. In humans, HSAs and closely related angiosarcomas are quite rare and similarly aggressive, with little known about their etiopathogenesis [5].

The PI3K/mTOR pathway is intimately associated with cell survival, proliferation, apoptosis, and cytoskeletal rearrangement. Activation of this pathway generally occurs through initial receptor tyrosine kinase activity, followed by a downstream phosphorylation cascade leading to the eventual phospho-activation of key pro-survival mediators, such as Akt [6]. This pathway has been shown to be dysregulated in many human cancer types including renal cell carcinoma, neuroendocrine tumors, and breast cancer [7]. Additionally, it appears to be constitutively activated in many canine cell lines, including canine mammary tumors, mast cell tumors, gliomas and HSA [8, 9].

The PI3K/mTOR pathway is also closely related to the vascular endothelial growth factor (VEGF) pathway [10–12]. Increased expression of the VEGF/VEGFR2 signaling pathway has been shown to be associated with increased proliferative activity in canine vascular tumors [13], and VEGFR2 is one of the upstream receptor tyrosine kinases known to signal through PI3K/Akt/mTOR [14]. Furthermore, upregulation of the VEGF pathway and increased VEGF expression has been shown to increase proliferation in hematologic malignancies [15].

In this study, we sought to examine the effect of PI3K/mTOR inhibition in canine HSA cell lines. We found that inhibition of this pathway decreased cell proliferation, increased apoptosis, decreased the ability of HSA cells to migrate and invade, and reduced VEGF production. Furthermore, inhibition of the PI3K/mTOR pathway demonstrated additive effects when combined with the standard of care cytotoxic drug, DOX.

## Materials and methods

### Cell lines and conditions

The cell lines included in the FACC Canine Tumor Cell Line panel are described in detail in a recent publication [16]. The DEN-HSA, SB-HSA, and CIN-HSA cell lines were established from dogs with spontaneously occurring HSA. The SB-HSA cell line was provided by Dr. Erin Dickerson (University of Minnesota) [17], and the CIN-HSA cell line was provided by Dr. Amy MacNeill (University of Illinois) [18]. The DEN-HSA cell line was developed in the laboratory of one of the Authors (DHT) [19]. All cell lines were serially passaged by trypsinization or density gradient centrifugation and maintained in complete Eagle’s minimal essential medium (EMEM, VWR International, Radnor, PA) supplemented with nonessential amino acids, penicillin/streptomycin, L-glutamine and 10% fetal bovine serum (FBS, Atlas Biologicals, Fort Collins, CO) (C/10). They were maintained in standard conditions (37°C in a humidified 5% CO_2_ atmosphere). All cell lines were mycoplasma tested, and confirmed to be unique and canine in origin by microsatellite PCR and a multiplex species-specific PCR technique as described [20].

### Chemicals and reagents

**Fig 1:**
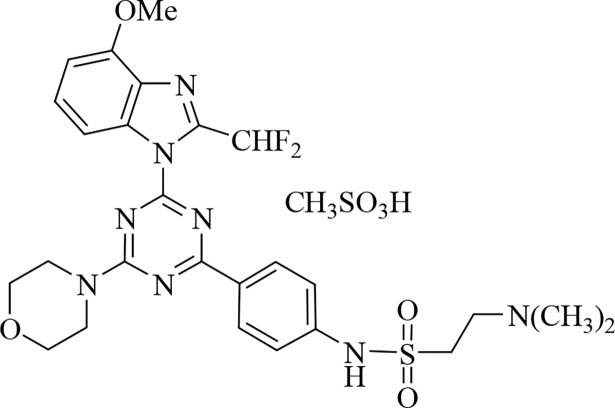
Chemical structure of VDC-597.

VDC-597 (Fig 1) is a dual-functioning inhibitor of PI3K alpha and mTORC1/2 with IC50 values of 19 nM and 14 nM for inhibition of human PI3K alpha and mTOR respectively in biochemical kinase assays, with approximately 10-fold less activity against the PI3K delta and gamma isoforms. VDC-597 has been profiled for kinase binding activity in kinomescan assays against 442 kinases. At a concentration of 1 uM, no significant binding to any of the kinases tested was observed, while at 10 uM, 5 of 442 kinases (DMPK2, EPHB6, MKNK1, MRCKA and TAOK1) showed weak binding. It has a molecular weight of 684.74 and has very good oral bioavailability in rodents (30–50%) and dogs (30–100%) (VetDC Inc, personal communication). VDC-597 is being developed by VetDC, Inc. (Fort Collins, CO) as a potential therapeutic candidate for veterinary cancer. It was supplied by VetDC as a dry powder, and 10 mM stock solution aliquots were made in DMSO and frozen until use. Doxorubicin USP was obtained from Bedford Laboratories (Bedford, OH). Mitomycin C was obtained from Sigma (St. Louis, MO).

### Viral transfection of cell lines

DEN-HSA and SB-HSA were virally transfected (according to the manufacturer’s directions) with NucLight Red Lentivirus Reagent (Essen Bioscience, Ann Arbor, MI), for nuclear labeling of cells with a red fluorescent protein to facilitate real-time microscopy. Puromycin selection was used to isolate the transfected population.

### Cell lysates

Cells were grown to approximately 70% confluence in C/10 and standard conditions and were washed with phosphate-buffered saline (PBS). The cells were then incubated for 24 hours in C/10 plus DMSO vehicle or varying concentrations of VDC-597 (0, 0.25, 0.5, 1.0, and 2.0 uM). Cells were washed again with PBS and lysed with M-PER mammalian protein extraction reagent (Thermo Scientific, Waltham, MA) containing 1 mM activated sodium orthovanadate, 1 mM phenylmethylsulfonyl fluoride in isopropanol, and protease inhibitor cocktail tablet (Complete Mini, Roche Diagnostics, Mannheim, Germany). Lysates were then homogenized using a 25-gauge needle, centrifuged, and supernatants were aliquoted and frozen at -80°C for storage. Total protein concentration was determined using a BCA Protein Assay Kit (Thermo Scientific).

### Western blot analysis

Lysates were heated to 95°C and supernatant was run on a 4–12% NuPAGE bis-tris precast gel in a NOVEX Xcell SureLock Mini-Cell System (Invitrogen, Carlsbad, CA) and transferred to a PVDF membrane (BioRad, Hercules, CA). The membrane was blocked using SuperBlock T20 Blocking Buffer (Thermo Scientific). Primary antibodies were applied and incubated overnight at 4°C (**Table 1**). Membranes were then washed using TBST and incubated with goat anti-rabbit horseradish peroxidase for 1 hour at room temperature in TBST. They were then developed using SuperSignal West Pico (Pierce, Rockford, IL) and bands visualized using a ChemiDoc XRS+ System (BioRad, Hercules, CA). Densitometric image analysis was then performed using ImageStudio Lite (Li-Cor Biosciences, Lincoln, NE).

**Table 1:** Antibodies utilized for western analysis.

Antibody	Catalog Number	Supplier	Dilution
Akt	9272	Cell Signaling Technology	1:1,000
pAkt	9271	Cell Signaling Technology	1:1,000
4EBP1	4923	Cell Signaling Technology	1:1,000
p4EBP1	2855	Cell Signaling Technology	1:1,000
GAPDH	ab37168	ABCAM	1:2,500
Goat α rabbit HRP	31460	Pierce	1:40,000

### Cell growth assay

Cells were seeded in quintuplicate at a concentration of 2,000 cells per well in 96-well plates with C/10 and adherent cells were allowed to adhere overnight. The following day, the plates were washed with phosphate-buffered saline (PBS) and replaced with fresh C/10 containing vehicle or C/10 with various concentrations of VDC-597 in quintuplicate. Suspension cells were treated immediately. In additional studies, HSA cells were plated in triplicate a concentration of 2,000 cells per well in a 96-well plate with C/10 EMEM and were allowed to adhere overnight. The following day, the plates were washed with PBS and replaced with fresh C/10, C/10 with various concentrations of DOX, or co-incubated in C/10 with combinations of VDC-597 and DOX together. The plates were then incubated for an additional 72 hours, followed by determination of relative viable cell number using a bioreductive fluorometric assay (Alamar Blue, AbD Serotec, Raleigh, NC) according to manufacturer’s recommendations using a Synergy HT microplate reader and associated KC4 software (Bio-Tek Instruments, Winooski, VT). Relative viable cell number was standardized to that of cells incubated in C/10 only and expressed as a percentage of that control. Each assay was repeated at least 3 times to insure reproducibility. The 50% growth inhibitory concentrations (IC50s) were calculated using nonlinear regression, fitting to a sigmoidal dose-response curve using a commercially available software program (Prism 7.0c, GraphPad Software, San Diego, CA).

### Cell growth and apoptosis assay

NucLight Red transfected cells were seeded in quintuplicate at a density of 2,000 cells/well in a 96 well plate and incubated overnight in standard conditions. Vehicle-containing C/10 or C/10 with various concentrations of VDC-597 plus YOYO®-1 iodide reagent (Life Technologies, Cat #: Y3601) at concentration of 50 nM (per manufacturer’s protocol) was added to the wells. The plate was placed within the Incucyte Live Cell Imaging apparatus (Essen BioScience, Ann Arbor, MI) and the number of viable cells (red fluorescing) and apoptotic cells (green fluorescing) were measured over time.

### Chemotaxis cell migration assay

The DEN-HSA cells were seeded in quintuplicate the top chamber of an IncuCyte® ClearView 96-Well Chemotaxis Plate at a density of 2,000 cells/well in EMEM containing 1% FBS (C/1) and varying concentrations of VDC-597. The bottom chamber of the plate was filled with EMEM containing 20% FBS (C/20) and varying concentrations of VDC-597. Migration of cells from top to bottom chamber through the plate’s micropore membrane was monitored via IncuCyte imaging and software analysis. The number of cells present in the bottom chamber was recorded and normalized to the initial number of cells present in the top chamber over time.

### Scratch wound cell migration assay

Cells were plated at a density of 25,000 cells/well in a 96-well ImageLock™ plate (Essen BioScience: 4379) and incubated overnight in standard conditions. A uniform defect (wound) was then created in the monolayer cells in the 96 well plate using the Essen BioScience WoundMaker™ and either mitomycin C (10 ug/mL) containing C/10 EMEM or mitomycin C (10 ug/mL) containing C/10 EMEM with various concentrations of VDC-597 was added. The plate was placed within the Incucyte apparatus in standard conditions and the relative wound density was recorded via IncuCyte imaging and software analysis over 48 hours.

### VEGF ELISA

Cells were plated at 100,000 cells/well in 12-well plates with C/10 for 24 hours under standard conditions. Cells were then washed and treated for 24 hours in C/10, or C/10 with various concentrations of VDC-597. The supernatant was then harvested and saved for VEGF quantification, 1 mL of fresh C/10 was then added to the plate, and the bioreductive method described above was used to determine relative viable cell number. The VEGF concentrations of the supernatant were evaluated using a canine ELISA in a 96-well format with components from R&D Systems (Minneapolis, MN). Specifically, this ELISA utilizes recombinant canine VEGF standards, a mouse anti-canine VEGF capture antibody, and a biotinylated goat anti-human VEGF detection antibody. Absorbances at 405 nm were determined using a Synergy HT microplate reader and KC4 software and results fitted to a 4-parameter logistic regression curve. The VEGF concentrations were standardized to relative viable cell number by expressing viable cell number as a percentage of control and dividing VEGF concentration by this percentage.

### Statistical analysis

Differences in number of viable cells for growth assay, wound density for scratch wound migration assay, normalized cells present in the bottom chamber for the chemotactic migration assay, and relative corrected VEGF concentrations under various VDC-597 concentrations were compared using nonparametric one-way analysis of variance (ANOVA) followed by a Tukey’s post-hoc test.

In order to determine the effects of addition of VDC-597 to DOX on viable cell number, the Bliss independence model was utilized [21]. Briefly, the Bliss criterion is described by the following equation:

E(x,y)=E(x)+E(y)–E(x)xE(y)

where E(x) is the fractional inhibition of concentration x of VDC-597 (between 0 and 1), E(y) is the fractional inhibition of concentration y of DOX, and E(x,y) is the combined effect. Theoretical growth inhibition curves were constructed using this equation, and standard deviations were estimated by error propagation of experimental SD. Differences between treatment groups (Bliss theoretical vs. experimental) were assessed using two-way ANOVA and a Bonferroni post-test. Using this model, if the experimental fractional inhibition across concentration was significantly higher or lower than the theoretical value, the interaction was considered synergistic or antagonistic respectively. If not significantly higher or lower, the interaction was considered additive. Statistical analyses were performed using Prism v7.0c. P values <0.05 were considered to be statistically significant.

## Results

### Proteins in the PI3K/mTOR pathway are activated and inhibitable in canine hemangiosarcoma cells

**Fig 2:**
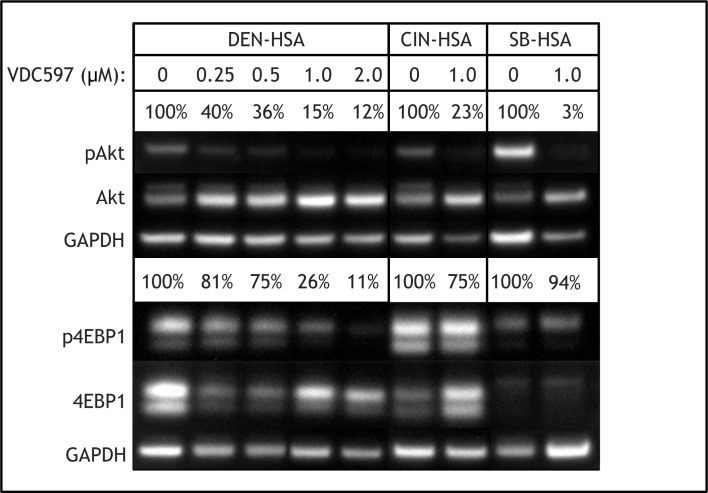
The dual PI3K/mTOR Inhibitor VDC-597 reduces downstream phosphorylation of Akt and 4eBP1 in canine hemangiosarcoma cells. DEN, CIN, and SB-HSA were incubated for 24 hours in standard conditions with either C/10 EMEM or C/10 EMEM with VDC-597 (range of concentrations for DEN-HSA, 1 uM for CIN- and SB-HSA), followed by protein extraction and western analysis. The addition of VDC-597 reduced downstream phosphorylation of both Akt and 4EBP1, as assessed by comparing the ratio of phospho-protein to total protein in each condition.

Western blot analysis demonstrated baseline phosphorylation of Akt and 4EBP1 in all three HSA cell lines, which was reduced with the addition of 1 uM VDC-597 (as assessed by determining the ratio of phosphorylated to total protein). Furthermore, Akt and 4EB1 phosphorylation was dose-dependently reduced by the addition of VDC-597 in DEN-HSA. (Fig 2; uncropped unmodified blots are included as S1 File). 50% inhibition of Akt phosphorylation was observed with a VDC-597 concentration of approximately 0.25 uM; however, 50% inhibition of the downstream target 4eBP1 was observed at a concentration of 0.5–1.0 uM.

### PI3K/mTOR inhibition reduces proliferation and induces apoptosis in canine hemangiosarcoma cells

**Fig 3:**
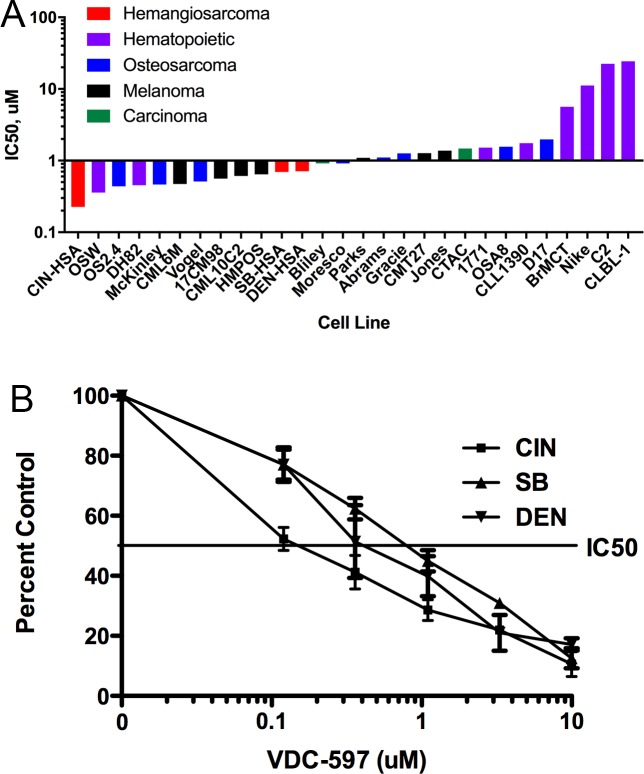
(A)50% Inhibitory concentrations following a 72-hour exposure to VDC-597 in canine tumor cell lines. Canine tumor cells were incubated in C/10 EMEM or C/10 EMEM with various concentrations of VDC-597 for 72 hours, followed by determination of relative viable cell number using a bioreductive fluorometric assay. IC50s were calculated using nonlinear regression, and presented values indicate means from 3 separate experiments. (B). VDC-597 Dose-Dependently Inhibits the Growth of Canine Hemangiosarcoma Cells. Canine HSA cells were exposed to varying concentrations of VDC-597 followed by determination of relative viable cell number as above. Calculated IC50s were 0.23, 0.69 and 0.71 uM for CIN-, SB-, and DEN-HSA respectively. Results shown indicate means of 3 separate experiments. Error bars indicate standard error measurements.

**Fig 4:**
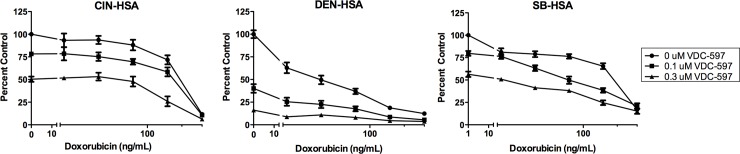
VDC-597 in combination with doxorubicin provides additive inhibitory effects on canine hemangiosarcoma cell growth. DEN, CIN, and SB-HSA cells were incubated in C/10 EMEM with varying concentrations of doxorubicin, or co-treated in C/10 EMEM with VDC-597 and doxorubicin for 72 hours followed by determination of relative viable cell number as above. Error bars indicate standard error measurements.

**Fig 5:**
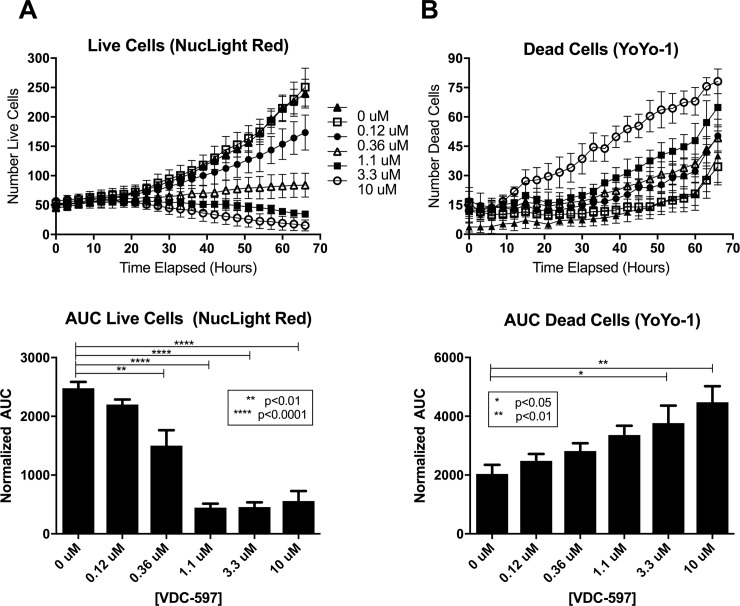
VDC-597 inhibits proliferation and induces apoptosis of canine hemangiosarcoma cells. NucLight Red-expressing SB-HSA cells were incubated for 72 hours with C/10 or C/10 with various concentrations of VDC-597 with the addition of YoYo-1 iodide reagent to label apoptotic cells. The number of viable (**A**) and number of apoptotic (**B**) cells per well were determined using an IncuCyte real-time videomicroscopy system. Area under the curve for each condition was calculated. Asterisks indicate *P* values less than 0.05. Error bars indicate standard error measurements.

Using a plate-based bioreductive fluorometric assay, we evaluated the growth inhibitory effects of VDC-597 against a previously described and characterized panel of canine tumor cell lines (16), with the addition of 2 canine HSA cell lines. After a 72-hour incubation period, IC50s ranged from 0.23 uM to >20 uM (Fig 3A). Interestingly, one of the canine HSA cell lines was the most sensitive of all the cell lines evaluated, and all three HSA cell lines demonstrated IC50s less than 1 uM. Growth inhibition curves for the three canine HSA cell lines are depicted in Fig 3B. Furthermore, the same assay was used to demonstrate an additive anti-proliferative effect when VDC-597 was combined with the current standard of care chemotherapeutic for canine HSA, DOX (Fig 4). Additionally, the IncuCyte Live Cell Imaging system was utilized to monitor both proliferation and apoptosis with the use of lentivirally transfected HSA cells expressing a red fluorescent nuclear protein and addition of YOYO®-1 iodide reagent to label apoptotic cells. The addition of VDC-597 dose dependently decreased proliferation and increased apoptosis in canine HSA cells (Fig 5). S2 File depicts time-lapse videomicroscopy of apoptosis of VDC-597 treated canine HSA cells.

### PI3K/mTOR inhibition reduces migration of canine hemangiosarcoma cells

**Fig 6:**
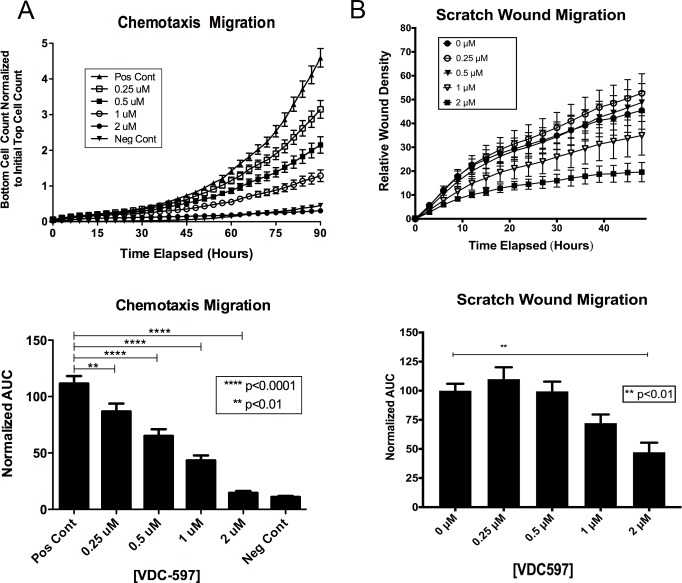
VDC-597 inhibits canine hemangiosarcoma cell migration. **A.** DEN-HSA was plated in the top chamber of a specialized 96-well chemotaxis migration plate in either C/0.1 EMEM or C/0.1 EMEM with various concentrations of VDC-597. C/20 EMEM or C/20 EMEM with identical various concentrations of VDC-597 of the top chamber was placed in the bottom chamber. Migration of the cells through the micropore membrane was assessed using the Incucyte Live Cell Imaging System over 90 hours. Area under the curve for each condition is expressed. Asterisks indicate *P* values less than 0.05. **B.** DEN-HSA was plated and allowed to grow to confluency in a specialized 96-well scratch wound assay plate. A uniform defect (“scratch”) was made through the confluent layer of cells and mitomycin C-containing C/10 EMEM with various concentrations of VDC-597 was added. Relative wound density was assessed using the Incucyte Live Imaging System over 48 hours. Area under the curve for each condition is expressed. Asterisks indicate *P* values less than 0.05. Error bars indicate standard error measurements.

Activation of the PI3K/mTOR pathway has been shown previously to promote cytoskeletal rearrangement and induce cell motility. To determine the effect of a dual inhibitor of this aspect of the pathway, two assays examining cell migration were explored. VDC-597 dose-dependently reduced migration capability of canine HSA cell lines in both a chemotactic (Fig 6A) and scratch wound assay (Fig 6B). S1 Fig depicts still images from the scratch wound assay.

### PI3K/mTOR inhibition reduces VEGF expression in canine hemangiosarcoma cells

**Fig 7:**
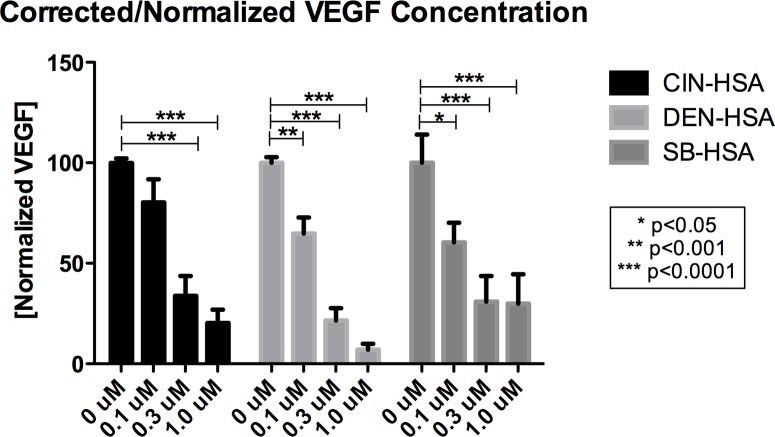
VDC-597 inhibits VEGF production in canine hemangiosarcoma cells. DEN, CIN, and SB-HSA cells were incubated in C/10 EMEM with various concentrations of VDC-597. 24 hours later, supernatants were harvested and utilized in a canine VEGF-specific ELISA. Relative viable cell number was concurrently assessed and VEGF concentration normalized to cell number. Error bars represent standard error measurements. Asterisks indicate *P* values less than 0.05.

The VEGF pathway has been shown to have associations with the PI3K/mTOR pathway. Being that HSA is an endothelial origin neoplasm, a reduction in expression of VEGF may have a significant impact on both autocrine signaling through expressed VEGFR2 and recruitment of “normal” blood vessels. An ELISA assay was utilized to assess the amount of VEGF produced in three HSA cell lines after 24 hours of treatment with various concentrations of VDC-597. A dose-dependent reduction in VEGF production (which was corrected for the number of viable cells present at the end of the assay period) was appreciated with the addition of VDC-597 (Fig 7).

## Discussion

Hemangiosarcoma is a malignant tumor of endothelial cells that is notoriously aggressive with regard to its metastatic tendencies. HSA is quite common in dogs, accounting for 5–7% of non-cutaneous primary tumors [22]. While surgery to remove the primary tumor is the initial treatment of choice (usually splenectomy), the median survival time (MST) with surgery alone is only approximately 2 months [23]. The addition of DOX based chemotherapy protocols increases the MST to 5–6 months, and although several other protocols have been examined, none have significantly improved survival time over others [24–26]. For this reason, investigations into additional molecular targets (particularly with regard to angiogenesis) to achieve a multimodal approach have been suggested [27].

While HSA is exceedingly rare in humans, the PI3K/Akt/mTOR pathway has also been shown to be upregulated in numerous human cancers [28], suggesting a potential for translational implications for studies performed with canine models. The benefits of using canine cancer as a human model have been well described, and include the spontaneous nature of the disease in dogs compared to induced models in rodents, the relatively rapid progression of disease compared to human cancer, and the aspect of shared environmental contributors among dogs and humans [29, 30]. Additionally, the lack of standard of care treatment protocols for many canine neoplasms allows for fast-tracked development of prospective trials, which often yield valuable information that could streamline progression of human clinical trials [30].

The PI3K/Akt/mTOR pathway has close association with receptor tyrosine kinases (RTKs), VEGF receptor pathways, p53, and PTEN, allowing for constitutive activation via many routes. Mutations involving RTK’s by mechanisms such as chromosomal translocations or regulatory region mutations, have been shown to constitutively activate the PI3K/mTOR pathway in malignancies like canine HSA [31]. VEGF receptor signaling is also a known stimulator of the PI3K/mTOR pathway, and when compared to normal human endothelial cells, human HSA cell lines demonstrate increased autocrine signaling and secretion of VEGF [32]. This paper further hypothesized that a potential source of the VEGF pathway activation involved mutations in the p53 gene. A 2014 study involving a conditional p53 knockout in hematopoietic and endothelial lineages in mice demonstrated a spontaneous development of HSA in 40% of knockout mice, and furthermore, that a single allelic deletion of VEGF was sufficient to reduce incidence of HSA to 8% [33]. Mutations in PTEN, a negative regulator of Akt signaling, have also been implicated in the development of multiple malignancies, and a 2005 study examining multiple canine HSA samples demonstrated a connection between mutations of PTEN, specifically at the C-terminus, and survival advantage in canine HSA. This study also suggested a link between loss of PTEN and VEGF expression, demonstrating that 8 of 12 HSAs with decreased PTEN had increased VEGF levels [34].

While activation of the PI3K/Akt/mTOR pathway in canine HSA has been verified and proven to be correlated with characteristics of malignancy [8, 35], there have been no studies to demonstrate functional effects of inhibition of this pathway in HSA. The PI3K/mTOR pathway is intimately involved with proliferation, apoptosis, migration, and angiogenesis, which makes it an ideal target, and while many drugs have been developed to inhibit the this pathway, they commonly target it at only one node [36]. This unilateral targeting has unfortunately been proven ineffective in many cases, and can even cause elevations in Akt activity (thus promoting proliferation and angiogenesis) due to the presence of negative feedback loops [37]. Dual inhibitors of this pathway have been suggested as having a therapeutic advantage not only to avoid the effects of negative feedback loops, but also to avoid the use of multiple separate drugs, which may have increased risk of adverse effects [38]. To date, there are no known studies to date that examine the effect of an inhibitor of this pathway in canine HSA.

In this study, we found that inhibition of the PI3K/Akt/mTOR pathway by VDC597 and resulted in decreased proliferation, increased apoptosis, reduced migration, and reduced VEGF expression in canine HSA cells. Importantly, the concentrations of VDC-597 associated with pathway inhibition and antitumor activity are predicted to be pharmacologically achievable, based on unpublished normal dog toxicokinetic studies and phase-I data in humans and dogs (VetDC, Inc., personal communication). It should be noted, however, that drug concentrations in excess of 1 uM are predicted to be supra-pharmacologic, and thus the results provided herein with higher VDC-597 concentrations should be interpreted with this caveat in mind.

The results observed here are consistent with one study performed with a monoinhibitor of mTOR (rapamycin) in human angiosarcoma, where pathway inhibition was found to reduce cell proliferation *in vitro* [39]. Multiple studies have been performed that examine Akt pathway inhibition in other human cancer types, including one study using a dual inhibitor of PI3K and mTOR in human head and neck cancer that demonstrated both *in vitro* and *in vivo* anti-neoplastic effects [40]. This study demonstrated that the dual inhibitor reduced proliferation by inducing cell cycle arrest and apoptosis and slowed growth of xenografted tumors. PI3/Akt/mTOR inhibitors have also been explored in other canine cancer types, with promising anti-neoplastic effects observed *in vitro* and in xenograft models in canine melanoma [41]. These results indicate that dual inhibition of the PI3K/mTOR pathway in canine HSA may provide beneficial *in vivo* anti-neoplastic effects and warrants further examination.

In conclusion, dual inhibition of Akt and mTORC1/2 with VDC-597 inhibited signaling through the PI3K/Akt/mTOR pathway, and inhibited multiple components of the malignant phenotype in canine HSA cells, including proliferation, migration, VEGF production, and DOX sensitivity, at concentrations predicted to be pharmacologically achievable. These results justify the evaluation of PI3K/mTOR inhibition as a therapeutic strategy in dogs with HSA.

## Supporting information

S1 Fig Still images depicting the scratch assay results depicted graphically in Fig 6B. (TIF)

S1 File Raw western images corresponding to the summary image in Fig 2. A key for the lane identifiers is included in the Word file within the folder of images.
(ZIP)

S2 File Time-lapse videomicroscopy of NucLight Red expressing DEN-HSA cells treated with 2 uM VDC-597. Apoptotic cells that have taken up the dye YoYo-1 demonstrate green fluorescence over 68 hours of incubation.
(MP4)

S3 File Raw data from proliferation assays. (XLSX)

## References

[1] Thamm DH. Hemangiosarcoma In: Withrow SJ, Vail DM, Page RL, editors. Small Animal Clinical Oncology. 5th ed Philadelphia: W. B. Saunders; 2012 p. 679–87.

[2] Lamerato-Kozicki AR, Helm KM, Jubala CM, Cutter GC, Modiano JF. Canine hemangiosarcoma originates from hematopoietic precursors with potential for endothelial differentiation. Exp Hematol. 2006;34(7):870–8. Epub 2006/06/27. 10.1016/j.exphem.2006.04.013 . 16797414

[3] Srebernik N, Appleby EC. Breed prevalence and sites of haemangioma and haemangiosarcoma in dogs. Vet Rec. 1991;129(18):408–9. Epub 1991/11/02. . 1767484 10.1136/vr.129.18.408

[4] Ogilvie GK, Powers BE, Mallinckrodt CH, Withrow SJ. Surgery and doxorubicin in dogs with hemangiosarcoma. Journal of Veterinary Internal Medicine. 1996;10(6):379–84. PubMed PMID: WOS:A1996VV31200007. 8947871 10.1111/j.1939-1676.1996.tb02085.x

[5] Cohen SM, Storer RD, Criswell KA, Doerrer NG, Dellarco VL, Pegg DG, et al Hemangiosarcoma in rodents: mode-of-action evaluation and human relevance. Toxicological Sciences. 2009;111(1):4–18. 10.1093/toxsci/kfp131 PubMed PMID: WOS:000269002200002. 19525443

[6] Vivanco I, Sawyers CL. The phosphatidylinositol 3-Kinase AKT pathway in human cancer. Nat Rev Cancer. 2002;2(7):489–501. Epub 2002/07/03. 10.1038/nrc839 . 12094235

[7] Porta C, Paglino C, Mosca A. Targeting PI3K/Akt/mTOR signaling in cancer. Front Oncol. 2014;4:64 Epub 2014/05/02. 10.3389/fonc.2014.00064 ; PubMed Central PMCID: PMCPMC3995050. 24782981 PMC3995050

[8] Murai A, Asa SA, Kodama A, Hirata A, Yanai T, Sakai H. Constitutive phosphorylation of the mTORC2/Akt/4E-BP1 pathway in newly derived canine hemangiosarcoma cell lines. BMC Vet Res. 2012;8:128 Epub 2012/07/31. 10.1186/1746-6148-8-128 ; PubMed Central PMCID: PMCPMC3438112. 22839755 PMC3438112

[9] Chen YT, Tan KA, Pang LY, Argyle DJ. The class I PI3K/Akt pathway is critical for cancer cell survival in dogs and offers an opportunity for therapeutic intervention. BMC Vet Res. 2012;8:73 Epub 2012/06/01. 10.1186/1746-6148-8-73 ; PubMed Central PMCID: PMCPMC3515332. 22647622 PMC3515332

[10] Karar J, Maity A. PI3K/AKT/mTOR Pathway in angiogenesis. Front Mol Neurosci. 2011;4:51 Epub 2011/12/07. 10.3389/fnmol.2011.00051 ; PubMed Central PMCID: PMCPMC3228996. 22144946 PMC3228996

[11] Hamada K, Sasaki T, Koni PA, Natsui M, Kishimoto H, Sasaki J, et al The PTEN/PI3K pathway governs normal vascular development and tumor angiogenesis. Genes Dev. 2005;19(17):2054–65. Epub 2005/08/19. 10.1101/gad.1308805 ; PubMed Central PMCID: PMCPMC1199575. 16107612 PMC1199575

[12] Young RJ, Fernando M, Hughes D, Brown NJ, Woll PJ. Angiogenic growth factor expression in benign and malignant vascular tumours. Exp Mol Pathol. 2014;97(1):148–53. Epub 2014/07/02. 10.1016/j.yexmp.2014.06.010 . 24984271

[13] Yonemaru K, Sakai H, Murakami M, Yanai T, Masegi T. Expression of vascular endothelial growth factor, basic fibroblast growth factor, and their receptors (flt-1, flk-1, and flg-1) in canine vascular tumors. Vet Pathol. 2006;43(6):971–80. Epub 2006/11/14. 10.1354/vp.43-6-971 . 17099154

[14] Adachi M, Hoshino Y, Izumi Y, Sakai H, Takagi S. Effects of inhibitors of vascular endothelial growth factor receptor 2 and downstream pathways of receptor tyrosine kinases involving phosphatidylinositol 3-kinase/Akt/mammalian target of rapamycin or mitogen-activated protein kinase in canine hemangiosarcoma cell lines. Can J Vet Res. 2016;80(3):209–16. Epub 2016/07/14. ; PubMed Central PMCID: PMCPMC4924555. 27408334 PMC4924555

[15] Podar K, Anderson KC. The pathophysiologic role of VEGF in hematologic malignancies: therapeutic implications. Blood. 2005;105(4):1383–95. Epub 2004/10/09. 10.1182/blood-2004-07-2909 . 15471951

[16] Fowles JS, Dailey DD, Gustafson DL, Thamm DH, Duval DL. The Flint Animal Cancer Center (FACC) canine tumour cell line panel: a resource for veterinary drug discovery, comparative oncology and translational medicine. Vet Comp Oncol. 2017;15(2):481–92. Epub 2016/05/21. 10.1111/vco.12192 . 27197945 PMC5656225

[17] Akhtar N, Padilla ML, Dickerson EB, Steinberg H, Breen M, Auerbach R, et al Interleukin-12 inhibits tumor growth in a novel angiogenesis canine hemangiosarcoma xenograft model. Neoplasia. 2004;6(2):106–16. Epub 2004/05/14. 10.1593/neo.03334 ; PubMed Central PMCID: PMCPMC1502086. 15140399 PMC1502086

[18] Tang L, Tong R, Coyle VJ, Yin Q, Pondenis H, Borst LB, et al Targeting tumor vasculature with aptamer-functionalized doxorubicin-polylactide nanoconjugates for enhanced cancer therapy. ACS Nano. 2015;9(5):5072–81. Epub 2015/05/06. 10.1021/acsnano.5b00166 . 25938427

[19] Thamm DH, Dickerson EB, Akhtar N, Lewis R, Auerbach R, Helfand SC, et al Biological and molecular characterization of a canine hemangiosarcoma-derived cell line. Res Vet Sci. 2006;81(1):76–86. Epub 2005/11/01. 10.1016/j.rvsc.2005.09.005 . 16256156

[20] O'Donoghue LE, Rivest JP, Duval DL. Polymerase chain reaction-based species verification and microsatellite analysis for canine cell line validation. J Vet Diagn Invest. 2011;23(4):780–5. Epub 2011/09/13. 10.1177/1040638711408064 . 21908323

[21] Zimmer A, Katzir I, Dekel E, Mayo AE, Alon U. Prediction of multidimensional drug dose responses based on measurements of drug pairs. Proc Natl Acad Sci U S A. 2016;113(37):10442–7. 10.1073/pnas.1606301113 ; PubMed Central PMCID: PMCPMC5027409. 27562164 PMC5027409

[22] Smith AN. Hemangiosarcoma in dogs and cats. Vet Clin North Am Small Anim Pract. 2003;33(3):533–52, vi. Epub 2003/07/11. . 12852235 10.1016/s0195-5616(03)00002-0

[23] Johnson KA, Powers BE, Withrow SJ, Sheetz MJ, Curtis CR, Wrigley RH. Splenomegaly in dogs. Predictors of neoplasia and survival after splenectomy. J Vet Intern Med. 1989;3(3):160–6. Epub 1989/07/01. . 2778749 10.1111/j.1939-1676.1989.tb03092.x

[24] Sorenmo KU, Jeglum KA, Helfand SC. Chemotherapy of canine hemangiosarcoma with doxorubicin and cyclophosphamide. J Vet Intern Med. 1993;7(6):370–6. Epub 1993/11/01. . 8114034 10.1111/j.1939-1676.1993.tb01033.x

[25] Dervisis NG, Dominguez PA, Newman RG, Cadile CD, Kitchell BE. Treatment with DAV for advanced-stage hemangiosarcoma in dogs. J Am Anim Hosp Assoc. 2011;47(3):170–8. Epub 2011/04/19. 10.5326/JAAHA-MS-5525 . 21498593

[26] Sorenmo KU, Baez JL, Clifford CA, Mauldin E, Overley B, Skorupski K, et al Efficacy and toxicity of a dose-intensified doxorubicin protocol in canine hemangiosarcoma. J Vet Intern Med. 2004;18(2):209–13. Epub 2004/04/03. . 15058772 10.1892/0891-6640(2004)18<209:eatoad>2.0.co;2

[27] Clifford CA, Mackin AJ, Henry CJ. Treatment of canine hemangiosarcoma: 2000 and beyond. J Vet Intern Med. 2000;14(5):479–85. Epub 2000/09/30. . 11012108 10.1892/0891-6640(2000)014<0479:tochab>2.3.co;2

[28] Courtney KD, Corcoran RB, Engelman JA. The PI3K pathway as drug target in human cancer. J Clin Oncol. 2010;28(6):1075–83. Epub 2010/01/21. 10.1200/JCO.2009.25.3641 ; PubMed Central PMCID: PMCPMC2834432. 20085938 PMC2834432

[29] Rowell JL, McCarthy DO, Alvarez CE. Dog models of naturally occurring cancer. Trends Mol Med. 2011;17(7):380–8. Epub 2011/03/29. 10.1016/j.molmed.2011.02.004 ; PubMed Central PMCID: PMCPMC3130881. 21439907 PMC3130881

[30] Vail DM, MacEwen EG. Spontaneously occurring tumors of companion animals as models for human cancer. Cancer Invest. 2000;18(8):781–92. Epub 2000/12/07. . 11107448 10.3109/07357900009012210

[31] Matsumura I, Mizuki M, Kanakura Y. Roles for deregulated receptor tyrosine kinases and their downstream signaling molecules in hematologic malignancies. Cancer Sci. 2008;99(3):479–85. Epub 2008/01/08. 10.1111/j.1349-7006.2007.00717.x . 18177485 PMC11158847

[32] Amo Y, Masuzawa M, Hamada Y, Katsuoka K. Expression of vascular endothelial growth factor in a human hemangiosarcoma cell line (ISO-HAS). Arch Dermatol Res. 2001;293(6):296–301. Epub 2001/08/02. . 11480589 10.1007/s004030100228

[33] Farhang Ghahremani M, Radaelli E, Haigh K, Bartunkova S, Haenebalcke L, Marine JC, et al Loss of autocrine endothelial-derived VEGF significantly reduces hemangiosarcoma development in conditional p53-deficient mice. Cell Cycle. 2014;13(9):1501–7. Epub 2014/03/15. 10.4161/cc.28474 ; PubMed Central PMCID: PMCPMC4050148. 24626176 PMC4050148

[34] Dickerson EB, Thomas R, Fosmire SP, Lamerato-Kozicki AR, Bianco SR, Wojcieszyn JW, et al Mutations of phosphatase and tensin homolog deleted from chromosome 10 in canine hemangiosarcoma. Vet Pathol. 2005;42(5):618–32. Epub 2005/09/08. 10.1354/vp.42-5-618 . 16145208

[35] Murai A, Abou Asa S, Kodama A, Sakai H, Hirata A, Yanai T. Immunohistochemical analysis of the Akt/mTOR/4E-BP1 signalling pathway in canine haemangiomas and haemangiosarcomas. J Comp Pathol. 2012;147(4):430–40. 10.1016/j.jcpa.2012.05.002 . 22789858

[36] Hay N. The Akt-mTOR tango and its relevance to cancer. Cancer Cell. 2005;8(3):179–83. Epub 2005/09/20. 10.1016/j.ccr.2005.08.008 . 16169463

[37] Harrington LS, Findlay GM, Lamb RF. Restraining PI3K: mTOR signalling goes back to the membrane. Trends Biochem Sci. 2005;30(1):35–42. Epub 2005/01/18. 10.1016/j.tibs.2004.11.003 . 15653324

[38] Chappell WH, Steelman LS, Long JM, Kempf RC, Abrams SL, Franklin RA, et al Ras/Raf/MEK/ERK and PI3K/PTEN/Akt/mTOR inhibitors: rationale and importance to inhibiting these pathways in human health. Oncotarget. 2011;2(3):135–64. Epub 2011/03/18. 10.18632/oncotarget.240 ; PubMed Central PMCID: PMCPMC3260807. 21411864 PMC3260807

[39] Bundscherer A, Vogt T, Kohl G, Landthaler M, Hafner C. Antiproliferative effects of rapamycin and celecoxib in angiosarcoma cell lines. Anticancer Res. 2010;30(10):4017–23. . 21036716

[40] Chang KY, Tsai SY, Wu CM, Yen CJ, Chuang BF, Chang JY. Novel phosphoinositide 3-kinase/mTOR dual inhibitor, NVP-BGT226, displays potent growth-inhibitory activity against human head and neck cancer cells in vitro and in vivo. Clin Cancer Res. 2011;17(22):7116–26. 10.1158/1078-0432.CCR-11-0796 . 21976531

[41] Wei BR, Michael HT, Halsey CH, Peer CJ, Adhikari A, Dwyer JE, et al Synergistic targeted inhibition of MEK and dual PI3K/mTOR diminishes viability and inhibits tumor growth of canine melanoma underscoring its utility as a preclinical model for human mucosal melanoma. Pigment Cell Melanoma Res. 2016;29(6):643–55. 10.1111/pcmr.12512 ; PubMed Central PMCID: PMCPMC5132162. 27463366 PMC5132162

