# Supplementary figures and images for: In vitro effects of PI3K/mTOR inhibition in canine hemangiosarcoma
Source: PLoS One. 2018 Jul 16;13(7):e0200634. doi: 10.1371/journal.pone.0200634 (PMC6047806; doi:10.1371/journal.pone.0200634)

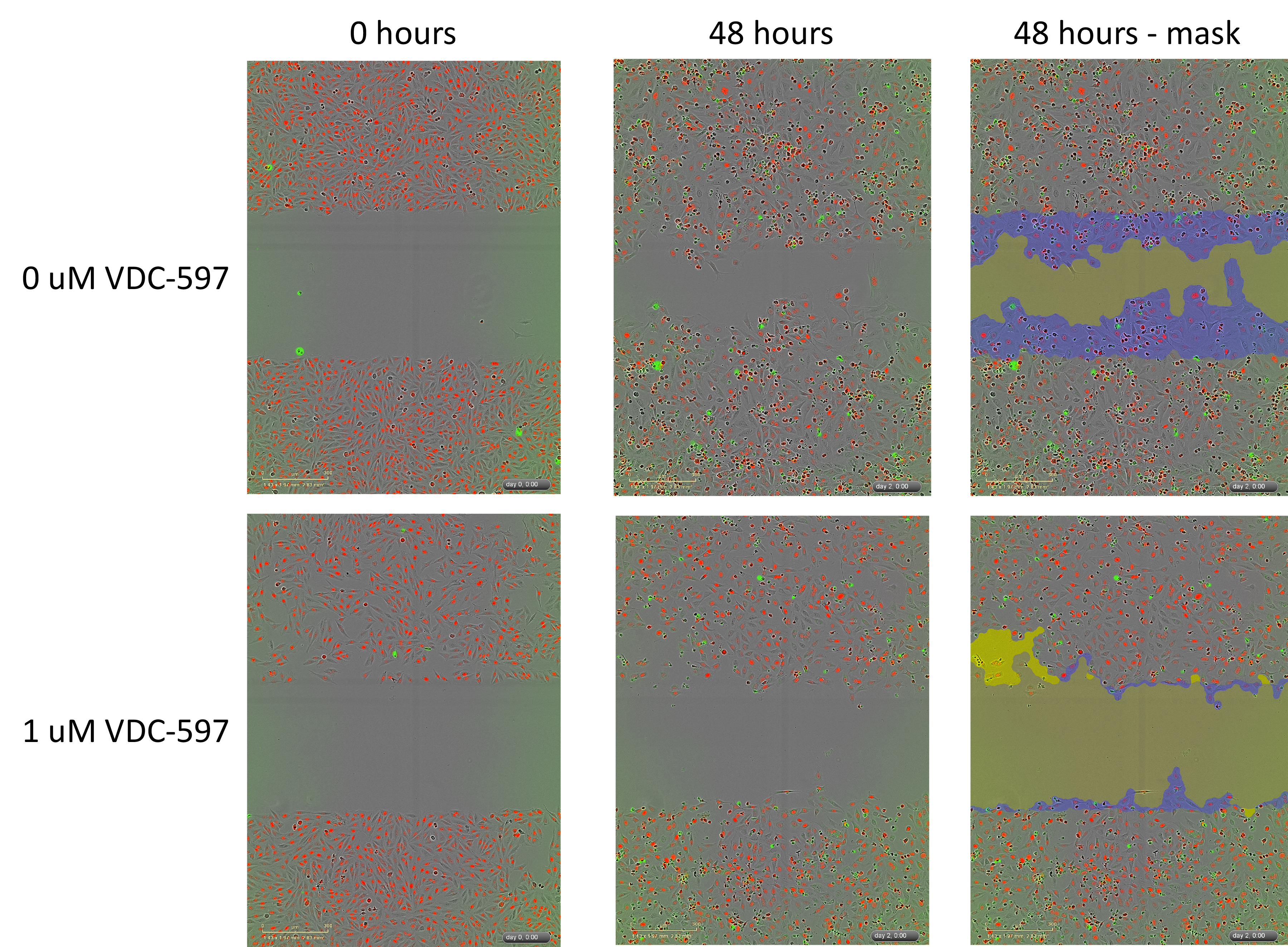

Supplement: S1 Fig — (TIF) [file pone.0200634.s001.tif]

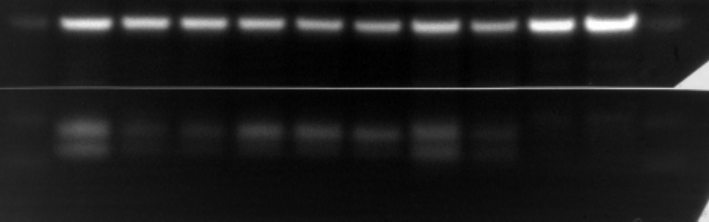

Supplement: S1 File — A key for the lane identifiers is included in the Word file within the folder of images. (ZIP) [file pone.0200634.s002.zip › Supplemental Data S1/Supplemental Data S1/4EBP1, GAPDH.tif]

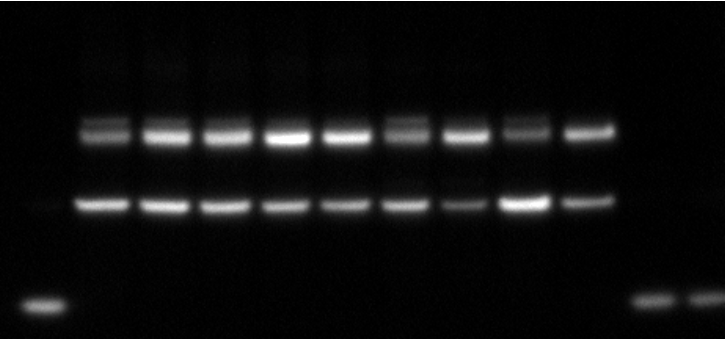

Supplement: S1 File — A key for the lane identifiers is included in the Word file within the folder of images. (ZIP) [file pone.0200634.s002.zip › Supplemental Data S1/Supplemental Data S1/AKT, GAPDH.tif]

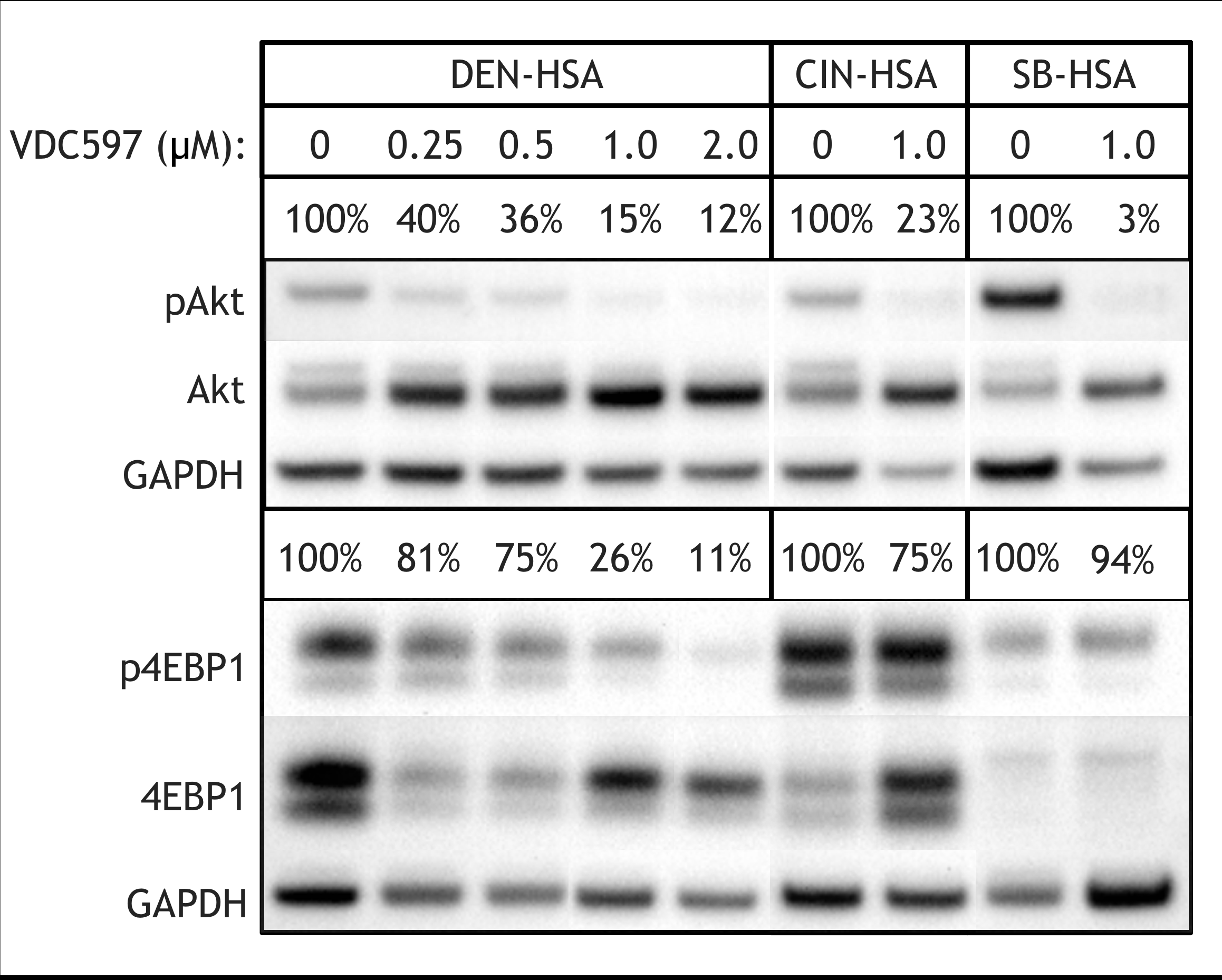

Supplement: S1 File — A key for the lane identifiers is included in the Word file within the folder of images. (ZIP) [file pone.0200634.s002.zip › Supplemental Data S1/Supplemental Data S1/HSA Densitometry repaired.tif]

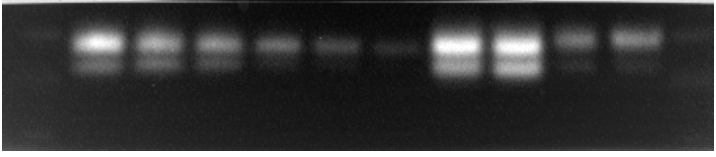

Supplement: S1 File — A key for the lane identifiers is included in the Word file within the folder of images. (ZIP) [file pone.0200634.s002.zip › Supplemental Data S1/Supplemental Data S1/p4EBP1.tif]

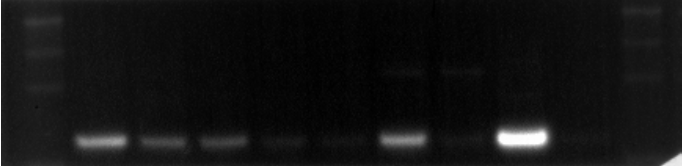

Supplement: S1 File — A key for the lane identifiers is included in the Word file within the folder of images. (ZIP) [file pone.0200634.s002.zip › Supplemental Data S1/Supplemental Data S1/pAKT.tif]
