# Supplementary material for: In vitro effects of PI3K/mTOR inhibition in canine hemangiosarcoma
Source: PLoS One. 2018 Jul 16;13(7):e0200634. doi: 10.1371/journal.pone.0200634 (PMC6047806; doi:10.1371/journal.pone.0200634)
Supplement: S1 File — A key for the lane identifiers is included in the Word file within the folder of images. (ZIP) [file pone.0200634.s002.zip › Supplemental Data S1/Supplemental Data S1/Lane identifier key for all western blots.docx]

Lane identifier key for AKT/GAPDH and pAKT western blots

1. Ladder
2. DEN-HSA, 0 uM VDC-597
3. DEN-HSA, 0.25 uM VDC-597
4. DEN-HSA, 0.5 uM VDC-597
5. DEN-HSA, 1.0 uM VDC-597
6. DEN-HSA, 2.0 uM VDC-597
7. CIN-HSA, 0 uM VDC-597
8. CIN-HSA, 1 uM VDC-597
9. SB-HSA, 0 uM VDC-597
10. SB-HSA, 1 uM VDC-597

Lane identifier key for 4eBP1/GAPDH and p4eBP1 western blots

1. Ladder
2. DEN-HSA, 0 uM VDC-597
3. DEN-HSA, 0.25 uM VDC-597
4. DEN-HSA, 0.5 uM VDC-597
5. DEN-HSA, 0.75 uM VDC-597
6. DEN-HSA, 1.0 uM VDC-597
7. DEN-HSA, 2.0 uM VDC-597
8. CIN-HSA, 1 uM VDC-597
9. CIN-HSA, 0 uM VDC-597
10. SB-HSA, 0 uM VDC-597
11. SB-HSA, 1 uM VDC-597

*Note that in the summary figure appearing in the manuscript (figure 2), the following alterations were made (affected lanes are in red above):*

Lane 5 was omitted and the lanes shifted left so there was alignment between the AKT and 4eBP1 gels

Lanes 8 and 9 were reversed in order to account for the inadvertent misloading of these lanes.
